# Physical performance tasks were linked to the PROMIS physical function metric in patients undergoing hemodialysis

**DOI:** 10.1016/j.jclinepi.2023.04.007

**Published:** 2023-07

**Authors:** Gregor Liegl, Felix H. Fischer, Mark Woodward, Marietta Török, Giovanni F.M. Strippoli, Jörgen Hegbrant, Andrew Davenport, Krister Cromm, Bernard Canaud, Michiel L. Bots, Peter J. Blankestijn, Claudia Barth, Kathrin I. Fischer, Matthias Rose

**Affiliations:** a Center for Patient-Centered Outcomes Research (CPCOR), Charité – Universitätsmedizin Berlin, corporate member of Freie Universität Berlin and Humboldt-Universität zu Berlin, Berlin, Germany; b The George Institute for Global Health, School of Public Health, Imperial College London, London, UK; c The George Institute for Global Health, University of New South Wales, Sydney, Australia; d Diaverum, Malmö, Sweden; e Department of Precision and Regenerative Medicine and Ionian Area (DiMePRe-J) University of Bari, Italy & School of Public Health, University of Sydney, Sydney, Australia; f Division of Nephrology, Department of Clinical Sciences, Lund University, Lund, Sweden; g UCL Department of Renal Medicine, Royal Free Hospital & University College London, London, UK; h Fresenius Medical Care Deutschland GmbH, Global Medical Office, Bad Homburg, Germany; i Montpellier University, School of Medicine, Montpellier, France; j Julius Center for Health Sciences and Primary Care, University Medical Center Utrecht, Utrecht University, Utrecht, The Netherlands; k Department of Nephrology & Hypertension, University Medical Center Utrecht, Utrecht, The Netherlands; l B. Braun Avitum AG, Medical Scientific Affairs, Melsungen, Germany

**Keywords:** Clinical outcome assessment, Physical function, Patient-reported outcomes, Performance outcomes, Linking, Hemodialysis

## Abstract

**Objectives:**

To investigate whether a multi-item performance outcome measure, the physical performance test (PPT), can be calibrated to a common scale with patient-reported outcome measures, using the Patient-Reported Outcomes Measurement Information System (PROMIS) physical function (PF) metric.

**Study Design and Setting:**

We analyzed baseline data (*N* = 1,113) from the CONVINCE study, an international trial in end-stage kidney disease patients comparing high-dose hemodiafiltration with high-flux hemodialysis. Assumptions of item response theory (IRT) modelling were investigated for the combined set of the nine-item PPT and a four-item PROMIS PF short form (PROMIS-PF4a). We applied unidimensional IRT linking for calibrating the PPT to the PROMIS PF metric.

**Results:**

Although some evidence for multidimensionality was found, classical test statistics (Cronbach's Alpha = 0.93), Mokken (Loevinger's *H* = 0.50), and bifactor analysis (explained common variance = 0.65) indicated that PPT and PROMIS-PF4a items can be used to assess a common PF construct. On the group level, the agreement between PROMIS-PF4a and linked PPT scores was stable across several subsamples. On the individual level, scores differed considerably.

**Conclusion:**

We found preliminary evidence that the PPT can be linked to the PROMIS PF metric in hemodialysis patients, enabling group comparisons across patient-reported outcome and performance outcome measures. Alternative linking methods should be applied in future studies using a more comprehensive PROMIS PF item set.

## Highlights

• PRO and PerfO items formed a psychometrically sound physical function scale.
• We found high potential for using a composite score of different assessment types.
• PerfO items were found to be suitable for calibration to the PROMIS PF metric.
• This study provides preliminary algorithms for linking PerfO items to PROMIS PF.

**What is new?**

**Key findings**
• The items of the PPT, which is a multi-item PerfO test battery, have the potential to be calibrated to the PROMIS PF metric, enabling group comparisons between PRO and PerfO measures of physical function in patients undergoing hemodialysis.

**What this adds to what was known?**
• The most frequently used assessment types for measuring physical function are PRO and PerfO. Although single-item PerfO measures have been shown to correlate poorly with PRO measures, recent studies found relatively high correlations between assessment types when multi-item PerfO test batteries were used. This study shows that a comprehensive PerfO measure with a broadly defined physical function constructs has the potential to be calibrated to a common scale with PRO measures using IRT modelling.

**What is the implication, what should change now?**
• The results of a unidimensional IRT-linking approach presented in this study can be used for comparing PPT data with PRO measures based on the standardized PROMIS PF metric. However, because the linking is based on only four PROMIS PF items, these results are preliminary and should be used with caution. Further studies based on a more comprehensive PROMIS PF item set should be carried out to apply and compare other linking methods, such as calibrated projection.

## 1 Introduction

As per the Patient-Reported Outcomes Measurement Information System (PROMIS) initiative, physical function is a broadly defined construct and can be specified as the ‘ability to carry out activities that require physical actions, ranging from self-care to more complex activities that require a combination of skills, often within a social context’ [1]. This generic definition of physical function is in line with the conceptual framework of the International Classification of Functioning, Disability, and Health by the World Health Organization [2]. Generic physical function and related constructs, such as the ability to participate in life, have been defined as core outcome domains in patients with end-stage kidney disease (ESKD) [[3], [4], [5]].

Besides laboratory measures of physiologic impairment (e.g., oxygen update or muscle loss) [6,7], patient-reported outcome (PRO) and performance outcome (PerfO) measures [8] are the most frequently used types of physical function assessment in ESKD patients [6]. Using PRO measures, patients rate their level of functioning by responding to questions or statements without interpretation by anyone else, usually in a paper-based or electronic questionnaire [8]. PRO measures allow for capturing a wide range of physical activities relevant to the patient's life. Therefore, PROs are particularly suitable for measuring generic physical function and have been explicitly recommended for outcome assessment in ESKD patients [3,4].

In contrast, PerfO measures assess a patient's performance on physical tasks based on standardized instructions by a test administrator in a standardized environment [8]. PerfO measures lead to more objective assessments than PROs, as they are less influenced by subjective patient variables that determine patient self-perception, including depression and pain [[9], [10], [11]]. However, PerfO assessments are resource-intensive and often applied as single-task measures (e.g., the chair stand test) or short test batteries focusing on narrowly specified subdomains of physical function (e.g., knee mobility).

Given these differences, it is not surprising that previous studies have mostly found weak to moderate correlations between typical PerfO and PRO measures [10,11]. As a consequence, it has been recommended that results should not be pooled or directly compared across assessment types because distinct constructs might be assessed [12]. This restricts the aggregation of research findings on physical function outcomes (e.g., by meta-analyses) within and across different medical fields. Nevertheless, some comprehensive PerfO test batteries have been developed, summarizing the performance of different physical tasks within a generic overall score [13]. Recent studies indicate that such multi-item PerfO measures with broadly defined underlying constructs might be suitable to measure the same physical function construct as generic PRO measures [9,14]. In that case, PerfO scores and PRO measures could be linked to a common metric, making scores directly comparable across different instrument types [15].

In the past decade, item response theory (IRT) modelling has increasingly been used to establish common metrics of different PRO measures assessing the same underlying construct [16,17]. As a prominent example, the PROMIS initiative provides a well-established common physical function metric, based on IRT calibration of a comprehensive PRO item bank [18]. Some widely used PRO measures of physical function have already been calibrated to the PROMIS physical function (PROMIS PF) metric, allowing for meaningful comparisons of scores across these instruments [17,19].

A recent study, based on a small sample of patients with different medical conditions, found that a generic PerfO battery, the physical performance test (PPT), might be sufficiently highly associated with PROMIS PF scores to be linked to the PROMIS PF metric using IRT methodology, with a Pearson correlation of 0.76 [9]. The purpose of the present study was to investigate whether the PPT can reliably be calibrated to the PROMIS PF metric in a large sample of ESKD patients treated by hemodialysis.

## 2 Materials and methods

### 2.1 The CONVINCE study

The CONVINCE study (Netherland Trial Register 7138) is the largest randomized controlled trial, to date, comparing efficacy and safety of high-flux hemodialysis vs. high-dose hemodiafiltration [20]. Participants were recruited in academic and hospital-based dialysis centers as well as in dialysis centers operated by private providers in eight countries from Eastern Europe (Hungary, Romania), Western Europe (France, Germany, the Netherlands, United Kingdom), and Southern Europe (Portugal, Spain). Inclusion criteria included being ≥ 18 years, diagnosed with ESKD, and undergoing hemodialysis treatment for ≥ 3 months [20]. Potentially eligible patients were given written information about the study and asked to sign an informed consent form. In addition to survival and cost-effectiveness, the CONVINCE study focusses on PROs, particularly on important aspects of health-related quality of life, such as physical function [20].

### 2.2 Measures

#### 2.2.1 The PROMIS physical function metric

The generic PROMIS physical function item bank (PROMIS PF) was originally developed to standardize patient-reported physical function across different populations and covers four subdomains: mobility, central regions (back/neck), upper extremity, and instrumental activities of daily living [18]. IRT calibration of the item bank using a graded response model (GRM) [21] enables item subsets to be used to assess an individual's ability level on a standardized T-score metric with a general population mean of 50 and a standard deviation of 10. Higher T-scores indicate better functioning. An advantage of IRT-calibrated item banks is that they can be extended by adding new items without changing the original metric. By doing so, other widely used physical function measures have already been calibrated to the PROMIS PF T-score metric, allowing for meaningful comparisons of scores across these instruments [17,19].

The PROMIS PF item bank version 1.2, consisting of 121 items with a five-point response scale, as well as several short forms have been translated into different languages, allowing for valid comparisons across countries [[22], [23], [24]]. In the CONVINCE study, patient-reported physical function was assessed with a PROMIS PF short form (PROMIS-PF4a), covering four instrumental activities of daily living and mobility items, at baseline and at several follow-ups [20]. Each PROMIS-PF4a language version used in CONVINCE has previously been translated using standardized guidelines as recommended by PROMIS [25].

#### 2.2.2 The physical performance test

The physical performance test (PPT) is a PerfO test battery consisting of nine physical tasks: (1) writing a sentence, (2) simulated eating, (3) lifting a book and putting it on a shelf, (4) putting on and removing a jacket, (5) picking up a coin from the floor, (6) turning 360°, (7) 50-foot walk test, (8) climbing one flight of stairs, and (9) climbing multiple flights of stairs [26]. The performance on each task is rated on a five-point response scale, mostly based on the time needed for performing a task. Overall PPT sum scores are calculated by adding up the individual scores for each task; higher scores indicating better functioning. A standardized test protocol, including guidance on test administration and scoring, is provided in English and has been translated into other languages using a standardized approach for use in the CONVINCE study. To ensure valid measurement results across countries and study sites, all test administrators were equipped with standardized PPT test kits and received standardized training. Like PROMIS PF, the PPT assesses a generic physical function construct and covers several subdomains. In the CONVINCE study, the PPT was only administered at baseline [20].

#### 2.2.3 Further measures

To investigate the differential association of patient-reported and performance-based physical function with depression and pain, the PROMIS depression four-item short form (higher scores indicate more depressive mood) and the PROMIS pain intensity single-item measure (higher scores indicate more severe pain) were applied [20].

### 2.3 Participants and sample size

For this study, baseline data from participants who answered all four items of the PROMIS-PF4a and at least six of nine PPT items were used. This procedure resulted in a sample size well above the recommended minimum requirements (*N* ≥ 500) for accurate GRM parameter estimates of items with five response options [27,28].

### 2.4 Statistical analysis

Psychometric analysis was conducted following the PROMIS analysis plan [29]. To provide initial descriptive information about the performance of the pooled set of PPT and PROMIS-PF4a items, internal consistency and corrected item-total correlations were calculated [[29], [30], [31]]. To determine the association between the PPT and the PROMIS-PF4a, the correlation between the latent variables underlying the PPT items and the PROMIS PF items, using structural equation modeling, was used.

Before calibrating the items of both measures to a common scale, assumptions of unidimensional IRT modeling were checked [32], including monotonicity, unidimensionality, and measurement invariance. Mokken analysis was used to check for monotonicity, meaning that subjects with higher ability levels are more likely to score higher on an item [33,34]. Loevinger's homogeneity coefficient *H* was calculated to determine scalability for the pooled item set and item-specific *H*_*j*_ values were calculated, indicating the discriminative power of individual items [33]. To evaluate unidimensionality, confirmatory factor analysis with a diagonally weighted least squares estimator was used [29,35]. Exploratory bifactor analysis with one general factor and four uncorrelated specific factors for each potential physical function subdomain was additionally used to evaluate ‘essential’ unidimensionality [29,36]. For the case of a low percentage of uncontaminated correlations (PUC < 0.8), an explained common variance > 0.6 and an *omegaH* value > 0.7 have been suggested as reasonable thresholds [36]; loadings on the general factor ≥ 0.30 were defined as salient [29]. Measurement invariance with respect to age, sex, medical condition, dialysis duration, and region was investigated using ordinal logistic regression for examining differential item functioning (DIF) [37].

To link the PPT items to the PROMIS PF T-score metric, a unidimensional GRM was fitted to the pooled set of items of both measures, with parameters of PROMIS-PF4a items fixed to the originally established PROMIS PF parameters [18] and with PPT item parameters freely estimated. The expected a posteriori method was used for estimating IRT-based scores (theta), which were subsequently transformed to T-scores (T-score = theta∗10 + 50). Item fit was evaluated using the generalized S-X^2^ statistic [38].

To evaluate whether the linking of the PPT to the PROMIS PF metric was successful, the agreement between PPT and PROMIS-PF4a T-scores was investigated in several subsamples using Bland-Altman plots and standardized mean differences (SMD) for paired samples [39,40]. SMDs of < 0.2, < 0.5, < 0.8, and ≥ 0.8 were interpreted as negligible, small, medium, and large effect sizes, respectively [41]. To determine differential associations of physical function scores with known predictors (i.e., pain and depression) by assessment type, Pearson correlation was used.

R version 3.6.2 was used for statistical analyses, including the R packages EFAtools, effsize, lavaan, lordif, mirt, mokken, and psych [31,34,35,40,[42], [43], [44], [45]].

## 3 Results

After exclusion participants for exceeding the item nonresponse criteria, 1,113 participants were included in data analyses. Sociodemographic and clinical characteristics of the sample are presented in Table 1.

**Table 1:** Summary statistics for the study population (*N* = 1,113)

Female; *n* (%)	418 (37.6)
Age in years; mean (SD)	61.8 (13.4)
Age in years; median (range)	63.0 (20–92)
Hemoglobin g/dL; mean (SD)	11.3 (1.2)
Calcium mg/dL; mean (SD)	8.9 (0.7)
Phosphate mg/dL; mean (SD)	5.0 (1.4)
Creatinine mg/dL; mean (SD)	8.4 (2.3)
Kt/V; mean (SD)	1.69 (0.49)
Dialysis duration in years; *n* (%)	
Less than 1	225 (20.2)
1 to 5	599 (53.8)
More than 5	286 (26.0)
Cause of ESKD; *n* (%)	
Arteriosclerosis	47 (4.2)
Autoimmune disease	29 (2.6)
Congenital kidney disease	17 (1.5)
Diabetes	230 (20.7)
Glomerulonephritis	232 (20.8)
Hypertension	161 (14.5)
Interstitial nephritis	106 (9.5)
Polycystic kidney disease	123 (11.1)
Other	108 (9.7)
Multiple	6 (0.5)
Unknown	54 (4.9)
Comorbid conditions; *n* (%)	
Diabetes	376 (33.8)
CVD	502 (45.1)
Cancer	150 (13.5)
COPD	86 (7.7)
Region; *n* (%)	
Eastern European	433 (38.9)
Western European	324 (29.1)
Southern European	356 (32.0)
Pain intensity^a^; mean (SD)	3.0 (2.8)
Depression^b^; mean (SD)	50.4 (9.0)

*Abbreviations*: COPD, chronic obstructive pulmonary disease; CVD, cardiovascular disease; ESKD, end-stage kidney disease; *n*, number; PF, physical function; PPT, physical performance test; PROMIS, Patient-Reported Outcomes Measurement Information System; SD, standard deviation.

a As measured by a single item with a 0 to 10 rating scale; higher scores indicate more severe pain.

b T-scores as measured with the PROMIS depression four-item short form; higher scores indicate more depressive mood.

### 3.1 Psychometric properties of the combined set of PRO and PerfO items

Table 2 provides an overview of psychometric properties of the pooled set of PROMIS-PF4a and PPT items that were investigated. Individual item characteristics are presented in Table 3.

**Table 2:** Psychometric properties of the combined set of PROMIS-PF4a and PPT items

Psychometric properties	Statistics/indices	Criterion	Results	
**Basic classic test theory statistics**				
Internal consistency	Cronbach's Alpha	≥ 0.80	Alpha = 0.93	
	Change of Cronbach's Alpha, if item omitted	≤ 0.00	Change ≤ 0.00 in 13 of 13 items	
Corrected item-total correlation	Correlation of an item with sum score of remaining items (r_itc_)	≥ 0.40	r_itc_ ≥ 0.40 in 13 of 13 items	
Association of PPT and PROMIS-PF4a	Latent correlation^a^	-	Correlation = 0.66	
**Monotonicity**				
Mokken scale analysis	Scalability of the total scale (*H*)	≥ 0.30	*H* = 0.50	
	Scalability of individual items (*H*_*i*_)	≥ 0.30	*H*_*i*_ ≥ 0.30 in 13 of 13 items	
	Graphical check whether ICCs are monotonically increasing	ICCs increasing in 13 of 13 items		
**Essential Unidimensionality**				
Exploratory bifactor analysis (four specific factors)	ECV	> 0.60	0.65	
	*omegaH*	> 0.70	0.77	
	PUC	> 0.80	0.77	
	Salient general factor loadings	≥ 0.30	Loadings ≥ 0.40 in 13 of 13 items	
Confirmatory factor analysis (unidimensional)			Standard (DWLS)	Robust (WLSMV)
	CFI	≥ 0.95	0.97	0.91
	TLI	≥ 0.95	0.96	0.89
	RMSEA	≤ 0.06	0.16	0.17
	SRMSR	≤ 0.08	0.12	0.12
	Sufficiently high standardized factor loadings	≥ 0.50	Loadings ≥ 0.50 in 12 of 13 items	
	Residual correlations of item pairs (r_Res_)	≤ 0.25	r_Res_ ≤ 0.25 in 99% of item pairs	
**Differential item functioning**				
Age (median split)	Nagelkerke's pseudo R^2^-change	≤ 3%	R^2^-change ≤ 3% in all items	
Female vs. male			R^2^-change ≤ 3% in all items	
Diabetes vs. no diabetes			R^2^-change ≤ 3% in all items	
CVD vs. no CVD			R^2^-change ≤ 3% in all items	
Region			R^2^-change ≤ 3% in 12 of 13 items	
Dialysis duration			R^2^-change ≤ 3% in all items	
**IRT model statistics**				
Unidimensional GRM fit	S-X^2^*P* value	≥ 0.001	*P* ≥ 0.001 in all PPT items	

*Abbreviations*: CFI, comparative fit index; CVD, cardiovascular disease; DWLS, diagonally weighted least squares; ECV, explained common variance; GRM, graded response model; H, Loevinger's Homogeneity coefficient; ICC, item characteristic curve; IRT, item response theory; PPT, physical performance test; PROMIS-PF4a, 4-item short form of the Patient-Reported Outcomes Measurement Information System physical function item bank; r_itc_, corrected item-total correlation; RMSEA, root mean square error of approximation; S-X^2^, generalized S-X^2^ item fit index; SRMSR, standardized root mean square residual; TLI, Tucker-Lewis index; WLSMV, weighted least squares mean-variance adjusted.

a Correlation of the latent variables underlying the PPT and the PROMIS items, using confirmatory factor analysis with two instrument-specific factors and a WLSMV estimator.

**Table 3:** Individual item characteristics based on the pooled set of PROMIS-PF4a and PPT items

Item		Corrected item-total correlation	Scalability	Loading on the general factor in the exploratory bifactor model	Factor loadings in 1-factor confirmatory factor analysis
**Item ID**	**Assessment type (origin)**	**r**_**itc**_	***H***_***i***_	***g***	***Λ***
PFA11	Patient-reported (PROMIS-PF4a)	0.68	0.50	0.53	0.82
PFA21	Patient-reported (PROMIS-PF4a)	0.71	0.52	0.57	0.84
PFA23	Patient-reported (PROMIS-PF4a)	0.72	0.53	0.56	0.88
PFA53	Patient-reported (PROMIS-PF4a)	0.74	0.56	0.60	0.89
PPT_1	Performance-based (PPT)	0.46	0.36	0.42	0.47
PPT_2	Performance-based (PPT)	0.51	0.38	0.50	0.56
PPT_3	Performance-based (PPT)	0.56	0.42	0.53	0.59
PPT_4	Performance-based (PPT)	0.66	0.48	0.60	0.67
PPT_5	Performance-based (PPT)	0.72	0.53	0.68	0.72
PPT_6	Performance-based (PPT)	0.66	0.54	0.63	0.81
PPT_7	Performance-based (PPT)	0.79	0.57	0.77	0.83
PPT_8	Performance-based (PPT)	0.79	0.58	0.86	0.82
PPT_9	Performance-based (PPT)	0.76	0.55	0.75	0.79

*Abbreviations*: H_i_, Loevinger's Homogeneity coefficient (on item level); PROMIS-PF4a, 4-item short form of the Patient-Reported Outcomes Measurement Information System physical function item bank; PPT, physical performance test; r_itc_, corrected item-total correlation; S-X^2^, generalized S-X^2^ item fit index.

Basic test theory statistics indicated high internal consistency of the combined 13-item scale including PRO and PerfO items. Mokken scale analysis supported good scalability and monotonicity of the pooled item set. The results of the traditional confirmatory factor analysis indicated some deviations from a strictly unidimensional structure, especially when a robust estimator was used. Moreover, the association between the PPT and the PROMIS-PF4a (*r* = 0.66) was somewhat lower than expected based on previous studies (*r* ≥ 0.75). However, standardized factor loadings were more than 0.50 in 12 of 13 items (with the exception of PPT_1 ‘writing a sentence’ with λ = 0.46) and residual correlations were less than 0.25 in 99% of item pairs, indicating that one factor explains most covariation across items. The results of the exploratory bifactor analysis indicated the existence of both subdomain-related and assessment type–related specific factors but also supported essential unidimensionality (Appendix Figure A1). As shown in Table 3, all 13 items showed salient loadings on the general factor. The explained common variance by the general factor was 65% and *omegaH* was 0.77.

Measurement invariance of all 13 items was supported by DIF analysis with respect to age, sex, medical condition, and dialysis duration. As for European regions, one individual item of the PPT (‘writing a sentence’) showed DIF, which had a negligible effect on physical function scores based on all items (data not shown).

### 3.2 IRT calibrations

When calibrating the PPT to the PROMIS PF metric using a unidimensional GRM, item fit of each PerfO task was supported (Table 2). Individual item parameters and fit statistics are presented in the Appendix Table A2. In only one item (PPT_1), the slope was significantly smaller than a = 1, indicating low discriminative power with regard to the PROMIS PF construct.

In several gender-specific, age-specific, region-specific, and condition-specific subsamples, high agreement between average PPT-based T-scores and PROMIS-PF4a T-scores was found. Each individual SMD was less than 2, indicating negligible effect sizes (Table 4). As much as 27% of the sample achieved the highest possible PROMIS-PF4a T-score compared with 2% in the PPT.

**Table 4:** Agreement of measures after unidimensional IRT linking with fixed PROMIS-PF4a item parameters

Statistics	PROMIS-PF4a	PPT
T-score mean (SD)	43.1 (8.8)	43.0 (9.5)
T-score range: min - max.	22.0–53.6	11.8–61.9
SMD [95% CI] (PROMIS-PF-4a vs. PPT)		
Full sample	−0.01 [−0.07; 0.04]	
Subsample: Female	0.11 [0.02; 0.19]	
Subsample: Male	−0.09 [−0.17; −0.02]	
Subsample: Age < median	0.15 [0.05; 0.24]	
Subsample: Age ≥ median	−0.14 [−0.22; −0.07]	
Subsample: Eastern European	−0.09 [−0.18; 0.01]	
Subsample: Western European	0.17 [0.08; 0.26]	
Subsample: Southern European	−0.10 [−0.19; −0.01]	
Subsample: With CVD	−0.05 [−0.13; 0.03]	
Subsample: Without CVD	0.02 [−0.06; 0.10]	
Subsample: With diabetes	−0.11 [−0.20; −0.02]	
Subsample: Without diabetes	0.04 [−0.03; 0.11]	
Subsample: With cancer	0.01 [−0.13; 0.15]	
Subsample: Without cancer	−0.02 [−0.08; 0.04]	

*Abbreviations*: CI, confidence interval; CVD, cardiovascular disease; max., maximum; min., minimum; PROMIS-PF4a, 4-item short form of the Patient-Reported Outcomes Measurement Information System physical function item bank; PPT, physical performance test; SMD, standardized mean difference for paired samples; SD, standard deviation.

**Fig. 1:**
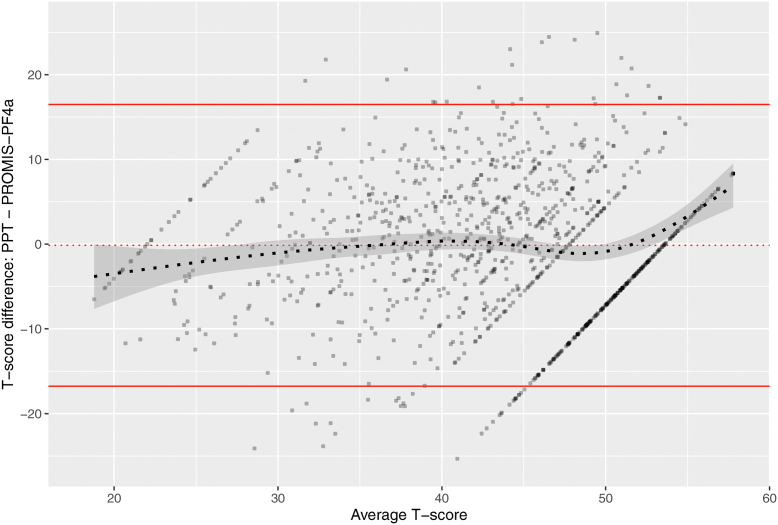
Bland-Altman plot showing the agreement of linked T-scores from both assessment types based on the unidimensional model with PROMIS-PF4a item parameters fixed. The dotted red line indicates the expected mean difference if there were perfect agreement. The bold red lines indicate 95% limits of agreement. The dotted black line indicates the expected mean difference and 95% confidence interval (grey area) at each average score. (For interpretation of the references to color in this figure legend, the reader is referred to the Web version of this article.)

**Fig. 2:**
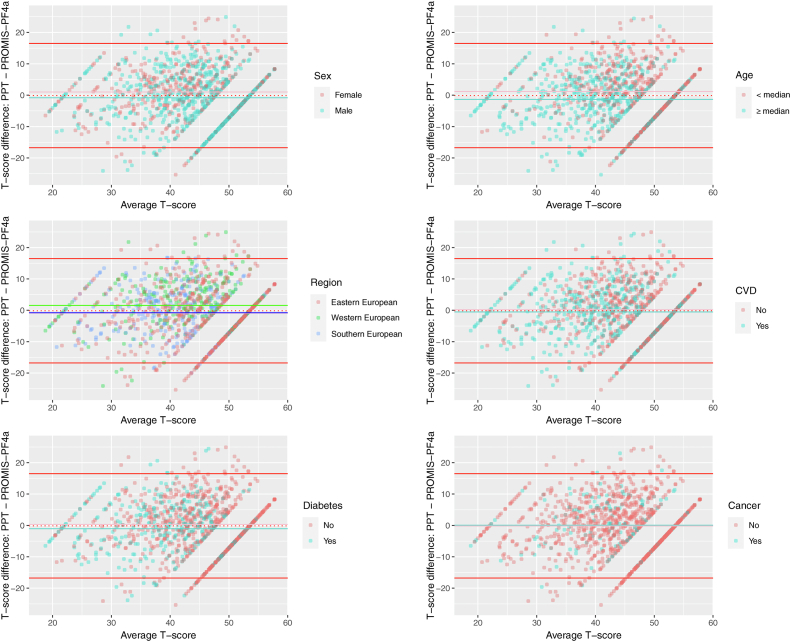
Bland-Altman plots showing the agreement of linked T-scores from both assessment types based on the unidimensional model with PROMIS-PF4a item parameters fixed for individual subsamples by sex, age, region, and medical conditions (cardiovascular disease, diabetes, and cancer). The colored lines as indicated by each plot's legend indicate the mean difference between PROMIS-PF4a and PPT scores for different subsamples. The bold red lines indicate 95% limits of agreement. The dotted red line indicates the expected mean difference if there were perfect agreement (0.0). (For interpretation of the references to color in this figure legend, the reader is referred to the Web version of this article.)

Bland-Altman plots supported high agreement between PROMIS-PF-4a and PPT T-scores at group level when unidimensional IRT linking was applied. Figure 1 shows that the agreement is largely stable along the entire T-score continuum. However, at the individual level, scores differed considerably, with up to 17 T-scores. Figure 2 shows that age, sex, regions, and comorbidities have a negligible effect on agreement between assessment types.

The differences between PPT and PROMIS-PF4a T-scores were slightly correlated with pain (*r* = 0.22) and depression (*r* = 0.28), indicating that more depressive mood and more pain intensity are associated with lower patient-reported compared to performance-based physical function (small effect sizes).

## 4 Discussion

Our findings indicate that performance-based and patient-reported measures can be used to assess a common physical function construct. The combined set of PPT and PROMIS-PF4a items formed a psychometrically sound scale for assessing generic physical function. On average, the patient-reported items loaded higher on the common construct, and so allowing more precise assessments. The PPT items, in contrast, allowed for a wider measurement range. These results are consistent with those of Kasper et al., who concluded that the use of a composite score of performance and self-report measures enables to combine the different strengths of both assessment types [14]. It should be noted that in our study, the PRO-based measurement range was particularly limited because only four PROMIS PF items were used. However, the appearance of ceiling and floor effects has also been discussed as a limitation of patient-reported physical function in general [46,47], highlighting the potential of using a composite score of different assessment types.

As psychometric requirements were met, we used unidimensional IRT modelling to calibrate the items of the PPT to the PROMIS PF metric. The corresponding linking results can be used for comparing PPT data with PROMIS PF measures and other PRO measures that have already been calibrated to the PROMIS PF metric [17,19]. To simplify the conversion of scores, the established PPT item parameters were incorporated into www.common-metrics.org. This website enables researchers to upload raw PPT data to obtain PROMIS PF T-scores [48]. Moreover, Appendix Table A3 provides a crosswalk table to directly transform PPT sum scores to PROMIS PF T-scores (and vice versa) [49]. It is important to note that these linking results should be considered preliminary at this stage and should be used with caution for two reasons. First, the correlation between PPT and PROMIS-PF4a scores was lower than expected based on previous research [9]. We can only speculate about the reasons for this. One plausible cause could be that more comprehensive PROMIS PF forms were used in previous studies, which covered a broader spectrum of physical activities and were therefore more similar to the PPT [9]. Second, for linking instruments that measure somewhat different latent constructs, other linking methods, such as calibrated projection, have been suggested [15]. However, calibrated projection is based on predicting scores of one measure from the scores of the other measure, which was not considered appropriate because of considerable ceiling effects indicated by PROMIS-PF4a. In sum, further studies using a more comprehensive PROMIS PF item set should be carried out to validate our findings.

As recommended for other linked measures that are not perfectly correlated [19], linked scores should only be compared at the group level. This is particularly important because PPT and PROMIS-PF4a T-scores differed substantially at the individual level. In contrast, the agreement between linked scores at the group level appeared to be stable across several subgroups by age, sex, medical condition, and region. These findings implicitly support the construct validity of PROMIS PF in ESKD patients undergoing hemodialysis, with performance-based physical function being a more objective criterion than PRO measures that were used before to investigate concurrent validity [23]. This suggests that, on group level, self-reports can be used to assess a generic physical function construct similar to more costly and burdensome PerfO test batteries.

This study has further limitations. First, consistent with previous studies [9,10], we found that the agreement between patient-reported and performance-based physical function was associated with self-reported pain and depression. Although the effect sizes appeared to be low, this may lead to biased results when the established linking algorithm is used in other samples, particularly when studying patient groups with more severe pain and/or depression. Second, measurement invariance across individual countries could not be checked because sample sizes of individual counties were too small. However, DIF analysis did not indicate culture-related or language-related bias across Eastern, Southern, and Western European countries. Third, our study is based on data from ESKD patients undergoing hemodialysis. It is yet to be shown that the established linking results can be generalized to other populations.

To the best of our knowledge, there are no previous studies demonstrating the possibility to convert patient-reported to performance-based physical function scores, and vice versa. Related findings will help facilitating interpretation, comparison, and pooling of research findings across studies that use different types of outcome assessments. We hope that this study will encourage researchers to try to replicate our findings in other settings and patient populations and to apply and compare other linking methods to optimize the comparability of PRO and PerfO assessments in the long term.
