# Supplementary material for: Physical performance tasks were linked to the PROMIS physical function metric in patients undergoing hemodialysis
Source: J Clin Epidemiol. 2023 Jul;159:128–38. doi: 10.1016/j.jclinepi.2023.04.007 (PMC10495039; doi:10.1016/j.jclinepi.2023.04.007)
Supplement: Appendix [file mmc1.pdf]

Appendix

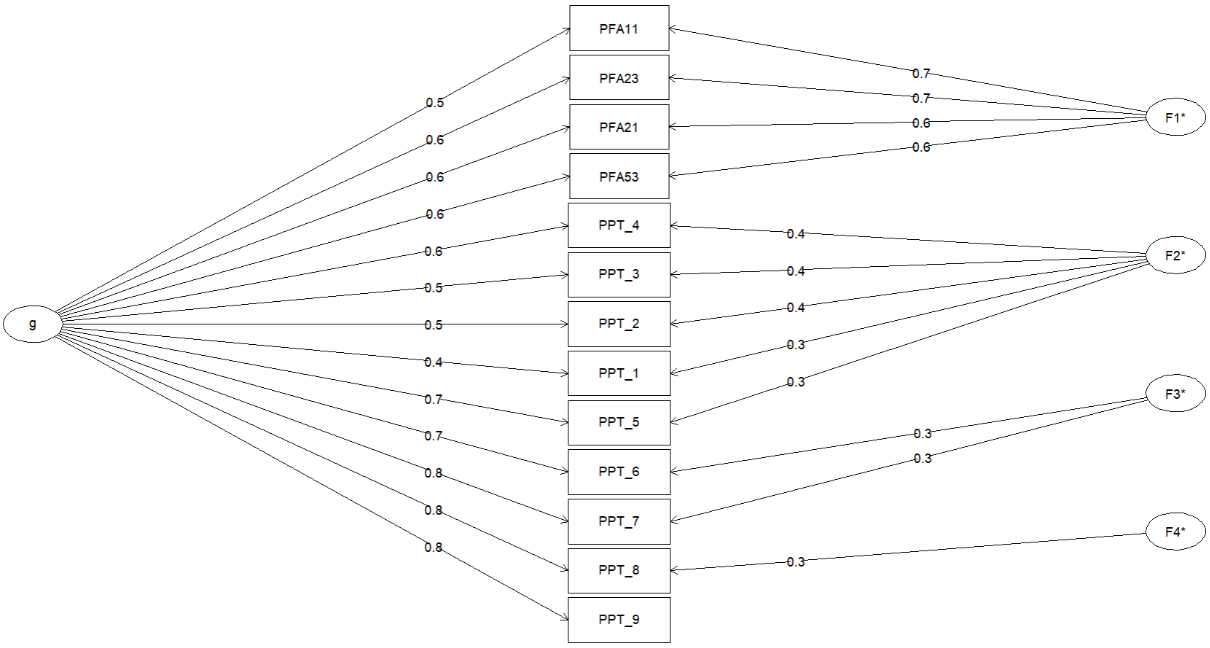

Figure A1: Loadings on the general physical function factor (g) and the specific factors (F1 to F4) in the exploratory bifactor analysis. The nine items of the PPT were assigned to subdomain-related factors (F2: upper extremity, F3: mobility/balance; F4: mobility/climbing stairs). All four PROMIS-PF4a items were assigned to F1. For all items of both measures, loadings on the general factor were ≥0.4.

*The specific factors are assumed to be uncorrelated. Loadings <0.2 are not shown.

Table A2: PPT item characteristics after linking to PROMIS PF using unidimensional IRT with fixed PROMIS-PF4a item parameters

Item		GRM fit	GRM item parameters [95% CI]*				
Item ID	Item description	$S\text{-}X^2$ p value	a	b1	b2	b3	b4
PPT_1	Writing a sentence	0.131	0.812 [0.671; 0.954]	-4.346 [-4.975; -3.717]	-1.506 [-1.703; -1.309]	-0.173 [-0.352; 0.005]	2.071 [1.574; 2.567]
PPT_2	Simulated eating	0.042	0.911 [0.763; 1.058]	-6.279 [-7.331; -5.226]	-2.929 [-3.287; -2.571]	-1.502 [-1.679; -1.326]	0.802 [0.536; 1.069]
PPT_3	Lifting a book & putting it on a shelf	0.027	0.940 [0.786; 1.095]	-4.873 [-5.572; -4.174]	-3.802 [-4.299; -3.306]	-2.570 [-2.877; -2.263]	-0.520 [-0.658; -0.382]
PPT_4	Putting on and removing a jacket	0.294	1.245 [1.079; 1.411]	-4.295 [-4.783; -3.806]	-2.415 [-2.638; -2.192]	-1.419 [-1.551; -1.287]	-0.122 [-0.250; 0.006]
PPT_5	Picking up a coin from the floor	0.268	1.528 [1.347; 1.709]	-2.871 [-3.110; -2.632]	-2.106 [-2.269; -1.942]	-1.289 [-1.393; -1.185]	0.433 [0.285; 0.582]
PPT_6	Turning 360 degrees	0.257	1.812 [1.531; 2.093]	-2.230 [-2.419; -2.040]	-1.729 [-1.862; -1.596]	-	-
PPT_7	50-foot walk test	0.136	1.870 [1.653; 2.086]	-3.024 [-3.256; -2.792]	-2.236 [-2.392; -2.081]	-1.693 [-1.808; -1.579]	-0.773 [-0.850; -0.696]
PPT_8	Climbing one flight of stairs	0.518	2.142 [1.880; 2.404]	-2.183 [-2.341; -2.025]	-1.571 [-1.684; -1.458]	-1.066 [-1.155; -0.978]	0.313 [0.184; 0.443]
PPT_9	Climbing multiple flights of stairs	0.309	2.323 [2.022; 2.623]	-2.021 [-2.164; -1.877]	-1.475 [-1.580; -1.369]	-0.991 [-1.076; -0.907]	-0.621 [-0.704; -0.538]

Abbreviations: IRT, item response theory; GRM, graded response model; PROMIS-PF4a, 4-item short form of the Patient-Reported Outcomes Measurement Information System physical function item bank; PPT, physical performance test; $S\text{-}X^2$, generalized $S\text{-}X^2$ item fit index

* *Note:* Item parameters are preliminary and should be used with caution

[a] For IRT-based scoring, the values of the original item PPT 6 (i.e., 0, 2, and 4) must be converted to 0, 1, and 2 to ensure that all categories have a distance of 1.

Table A3: Crosswalk table between PPT sum scores and PROMIS T-scores*

PPT sum score[a]	PROMIS PF T-score	SE	95% CI
0	12	5.5	1; 22
1	14	5.2	3; 24
2	16	5.0	6; 25
3	17	4.8	8; 27
4	19	4.7	10; 28
5	20	4.5	12; 29
6	22	4.4	13; 30
7	23	4.3	15; 31
8	24	4.2	16; 33
9	26	4.1	18; 34
10	27	4.0	19; 35
11	28	3.9	20; 36
12	29	3.9	22; 37
13	30	3.8	23; 38
14	31	3.8	24; 39
15	33	3.8	25; 40
16	34	3.8	26; 41
17	35	3.8	27; 42
18	36	3.8	28; 43
19	37	3.8	29; 44
20	38	3.8	30; 46
21	39	3.9	31; 47
22	40	4.0	33; 48
23	42	4.0	34; 49
24	43	4.1	35; 51
25	44	4.2	36; 52
26	45	4.3	37; 54
27	47	4.4	38; 56
28	49	4.6	40; 57
29	50	4.7	41; 60
30	52	4.9	43; 62
31	54	5.1	44; 64
32	56	5.2	46; 67
33	59	5.5	48; 70
34	62	5.8	51; 73

* *Note:* Linking results are preliminary and should be used with caution.

[a] For IRT modelling, the values of the original item PPT 6 (i.e., 0, 2, and 4) had to be converted to 0, 1, and 2 to ensure that all categories have a distance of 1. Consequently, the maximum total score is 34 instead of 36.
